# The relationship between combined antipsychotic use and clinical features in schizophrenia spectrum patients treated in inpatient ward: A retrospective study

**DOI:** 10.1192/j.eurpsy.2021.1383

**Published:** 2021-08-13

**Authors:** U. Baklaci

**Affiliations:** Psychiatry, Ege University Medical Faculty, Izmir, Turkey

**Keywords:** psychosis, antipsychotic, clozapine, combined antipsychotic

## Abstract

**Introduction:**

The combination of antipsychotics can be seen in up to 70% due to the presence of resistance to treatment, aggression, sleep disorders, and self destructive behavior in psychosis spectrum disorders in clinical practice. More side effects were observed in patients using antipsychotic combinations.

**Objectives:**

The aim of this study is retrospectively investigate the sociodemographic and clinical characteristics differences between antipsychotic combination and monotherapy groups

**Methods:**

The files of 754 cases admitted to the hospital from the first day of January 2013 to the last day of December 2016 were reached. Patients diagnosed as according to DSM-5 “Schizophrenia Spectrum and Other Psychotic Disorders” were included. From the files of these cases, sociodemographic characteristics, disease characteristics and antipsychotic properties (clozapine use, combined antipsychotic and depot antipsychotic use) were used. Pearson chi-square test and student t test were used in data analysis

**Results:**

Age was significantly lower in patients treated with combined antipsychotics than patients receiving monotherapy (t = 2,264, p = 0.026). Age of onset of psychosis was significantly lower in patients treated with combined antipsychotics (t = 2,771, p = 0.007). Education level was also found to be lower in this group (t = 2,333, p = 0.02) The duration of hospitalization was longer in patients treated with combined antipsychotics (t = 3,069, p = 0.002).

**Conclusions:**

There were statistically significant differences between the patients treated with combined antipsychotics compared to the group treated with monotherapy. These are the differences in the age of onset of psychosis, education level and duration of hospitalization.

